# Value-of-Information Analysis for External Validation of Risk Prediction Models

**DOI:** 10.1177/0272989X231178317

**Published:** 2023-06-22

**Authors:** Mohsen Sadatsafavi, Tae Yoon Lee, Laure Wynants, Andrew J Vickers, Paul Gustafson

**Affiliations:** Respiratory Evaluation Sciences Program, Faculty of Pharmaceutical Sciences, University of British Columbia, Vancouver, British Columbia, Canada; Respiratory Evaluation Sciences Program, Faculty of Pharmaceutical Sciences, University of British Columbia, Vancouver, British Columbia, Canada; Department of Epidemiology, CAPHRI Care and Public Health Research Institute, Maastricht University, Maastricht, The Netherlands; Department of Development and Regeneration, KU Leuven, Leuven, Belgium; Department of Epidemiology and Biostatistics, Memorial Sloan Kettering Cancer Center, New York, New York, USA; Department of Statistics, University of British Columbia, Vancouver, British Columbia, Canada

**Keywords:** predictive analytics, precision medicine, decision theory, value of information, bayesian statistics

## Abstract

**Background:**

A previously developed risk prediction model needs to be validated before being used in a new population. The finite size of the validation sample entails that there is uncertainty around model performance. We apply value-of-information (VoI) methodology to quantify the consequence of uncertainty in terms of net benefit (NB).

**Methods:**

We define the expected value of perfect information (EVPI) for model validation as the expected loss in NB due to not confidently knowing which of the alternative decisions confers the highest NB. We propose bootstrap-based and asymptotic methods for EVPI computations and conduct simulation studies to compare their performance. In a case study, we use the non-US subsets of a clinical trial as the development sample for predicting mortality after myocardial infarction and calculate the validation EVPI for the US subsample.

**Results:**

The computation methods generated similar EVPI values in simulation studies. EVPI generally declined with larger samples. In the case study, at the prespecified threshold of 0.02, the best decision with current information would be to use the model, with an incremental NB of 0.0020 over treating all. At this threshold, the EVPI was 0.0005 (relative EVPI = 25%). When scaled to the annual number of heart attacks in the US, the expected NB loss due to uncertainty was equal to 400 true positives or 19,600 false positives, indicating the value of further model validation.

**Conclusion:**

VoI methods can be applied to the NB calculated during external validation of clinical prediction models. While uncertainty does not directly affect the clinical implications of NB findings, validation EVPI provides an objective perspective to the need for further validation and can be reported alongside NB in external validation studies.

**Highlights:**

- External validation is a critical step when transporting a risk prediction model to a new setting, but the finite size of the validation sample creates uncertainty about the performance of the model.
- In decision theory, such uncertainty is associated with loss of net benefit because it can prevent one from identifying whether the use of the model is beneficial over alternative strategies.
- We define the expected value of perfect information for external validation as the expected loss in net benefit by not confidently knowing if the use of the model is net beneficial.
- The adoption of a model for a new population should be based on its expected net benefit; independently, value-of-information methods can be used to decide whether further validation studies are warranted.

When transporting a risk prediction model from one population to a new, plausibly related population, differences in settings, time, or place might affect its performance. This entails validating the model in an independent sample obtained from the new population.^1^ During such external validation, model performance is typically examined in terms of calibration, discrimination, and net benefit (NB).^2^ Calibration refers to the degree that predicted and actual risks agree and is typically evaluated using calibration plots.^3^ Discrimination quantifies the degree by which the model can separate low-risk from high-risk individuals and is classically quantified through the c-statistic.^2^ The NB is a decision-theoretic metric that considers the benefits and harms associated with risk stratification and is calculated via decision curve analysis.^4^ Due to its deep roots in decision theory as well as its ease of calculation, the NB approach has become a widely used tool for the evaluation of prediction models.

Given the finite size of the external validation sample, the assessment of the performance of a risk prediction model is accompanied by uncertainty. This is typically approached as a statistical inference problem (e.g., by presenting error bands around the calibration plot or 95% confidence interval [CI] around the c-statistic). Recent work on power and sample size calculations for external validation studies propose targeting prespecified standard errors on mean calibration, calibration slope, c-statistic, and NB.^5^ However, the use of standard inferential methods to express the uncertainty of a decision-theoretic measure is questionable.^6,7^

As risk prediction models are ultimately used to inform patient management, uncertainty in their performance can be assessed in terms of its impact on the outcomes of medical decisions. From this perspective, the finite size of the validation sample can lead to incorrect conclusions, for example, recommending the use of the model where in fact the best strategy is to treat all eligible patients. Thus, conclusions based on a finite validation sample can be associated with loss of clinical utility. Value-of-information (VoI) analysis is a set of concepts and methods rooted in decision theory that aims at quantifying the expected loss due to uncertainty in decisions, which can in turn inform the value of future research toward reducing uncertainty.^8^ In a recent work, we applied VoI analysis to the development phase of risk prediction models.^9^ We defined the expected value of perfect information (EVPI) for model development as the expected loss in NB due to uncertainty in the coefficients of a prediction model developed based on a finite sample. In this article, we extend such a concept from the development to the validation phase and propose the validation EVPI as the expected loss in clinical utility due to uncertainty about the NB of the model in a new population inferred from a finite validation sample.

## Context

We focus on a previously developed risk prediction model for a binary outcome that is now undergoing external validation in a new target population. We have access to a representative sample of n subjects from this new population (the validation sample). In this context, the model can be seen as a deterministic function π(X) that maps the covariate pattern X to predicted risk π for a binary outcome Y (e.g., risk of in-hospital mortality due to sepsis or experiencing an asthma attack in the next 12 mo). If there are other competing models during external validation, they can also be considered in this framework. However, without loss of generality and for the sake of brevity, in what follows we assume only 1 model is being assessed.

We focus on the primary goal of validation: whether the decision to use a prespecified model in this population provides clinical utility. We are interested in evaluating the expected loss in clinical utility due to the finiteness of the validation sample and the potential incorrect decision that such uncertainty might lead to (e.g., recommending to use the model whereas the most efficient decision is to treat all). We note that sometimes the investigator is not just interested in this pursuit but also whether the model needs updating in this population to improve its performance. The VoI analysis for such model revision is closely connected to the previously discussed development EVPI^9^ and hence is not the focus of this paper.

The measure of clinical utility that we will focus on is NB.^4^ In brief, to turn a continuous predicted risk to a binary classification (e.g., low versus high risk) to inform a treatment decision, a decision maker needs to specify a context-specific risk threshold z (in [0,1]).^10^ The risk threshold *z* is external to NB calculations and should be decided after considering the benefit/risk balance of the treatment that will follow in the context of stakeholders’ preferences.^10^ Treatment is offered to patients whose predicted risk is above this threshold. If the predicted risk is precisely at this threshold, the decision maker would be ambivalent between treatment and no treatment. Such ambivalence implies that the decision maker assigns a relative weight of z/(1−z) to each false-positive classification compared with each true-positive classification.^4^ Vickers and Elkin^4^ showed that such a weight can be used as an exchange rate between false and true positives (similar to the role that willingness to pay plays as an exchange rate between costs and effectiveness in economic evaluations). Consequently, the NB of the model at threshold z , compared with treating no one, can be calculated in net true-positive units as:

$$NB_{\mathrm{model}}=P_{\mathrm{True}\;\mathrm{Positive}}\left(z\right)-P_{\mathrm{False}\;\mathrm{Positive}}\left(z\right)\frac{z}{1-z}.$$

For brevity, we drop the notation that indicates the left side is dependent on z . Here, $P_{\mathrm{True}\;\mathrm{Positive}}\left(z\right)=P\left(\pi \geq z,Y=1\right)$ and $P_{\mathrm{False}\;\mathrm{Positive}}\left(z\right)=P\left(\pi \geq z,Y=0\right)$ . A consistent estimator of this NB in the sample is

$${\hat{\mathrm{NB}}}_{\mathrm{model}}=\frac{1}{n}\sum_{i=1}^{n}I\left(\pi_{i}\geq \;z\right)\left\{Y_{i}-\left(1-Y_{i}\right)\frac{z}{1-z}\right\}.$$

The strategy of using the model for patient management competes with at least 2 “default” strategies: treating no one and treating all. The NB of the former is zero by definition. The NB of treating all is

$$NB_{\mathrm{all}}=P_{0}-\left(1-P_{0}\right)\frac{z}{1-z},$$

where $P_{0}$ is the outcome prevalence in the population. $NB_{\mathrm{all}}$ can be consistently estimated in the sample as

$${\hat{\mathrm{NB}}}_{\mathrm{all}}=\frac{1}{n}\sum_{i=1}^{n}\left\{Y_{i}-\left(1-Y_{i}\right)\frac{z}{1-z}\right\}.$$

As is implicit in NB calculations, we assume the decision maker is risk-neutral and the only source of evidence is the validation sample at hand. Under such assumptions, the model is recommended if the sample value of its incremental utility over the default decisions,

$$d\hat{\mathrm{NB}}={\hat{\mathrm{NB}}}_{\mathrm{model}}-\max\left\{0,{\hat{\mathrm{NB}}}_{\mathrm{all}}\right\},$$

is positive.

## Motivating Example: Prediction of Mortality after Acute Myocardial Infarction (AMI)

Identifying the risk of immediate mortality after an AMI can enable stratification of more aggressive treatments for high-risk individuals. GUSTO-I was a large clinical trial of multiple thrombolytic strategies for AMI.^11^ This data set has frequently been used to study methodological aspects of developing or validating risk prediction models.^12–14^ In line with a previous study, we used the non-US sample of GUSTO-I (*n* = 17,796) as a sample from the development population to fit a prediction model for 30-day post-AMI mortality^12,15^. We are interested in externally validating this model for the US population and thus used the US sample (*n* = 23,034) for external validation. Such a validation sample is larger than typical sizes of samples in most practical contexts. To make a case for our developments, we assume that we have access to data for only 500 patients; we will later use the entire sample to study how EVPI changes with sample size. We randomly selected, without replacement, 500 individuals from the US sample to create such an exemplary validation data set. Because of the strict trial protocol, the US and non-US samples can be more similar than the development and validation samples in a typical external validation study. However, in simulation studies, we create more divergent simulated samples to study how EVPI behaves. Thirty-day mortality was 7.0% in the non-US sample, 6.8% in the entire US sample, and 8.6% in the validation sample. As in previous case studies using these data,^9^ our primary threshold of risk interest is 0.02, above which more aggressive thrombolytic treatments are justified. All analyses were conducted in the R statistical programming environment.^16^ Ethics approval was not required because the anonymized data are publicly available for research.

Our candidate risk prediction model is similar to the previously developed ones using this data set.^9^ We did not apply any shrinkage given the large development sample. The final model for 30-day post-AMI mortality based on applying logistic regression to the entire development sample was

$$\begin{array}{cc} & \mathrm{logit}\left(\pi \right)=-2.084+0.078*\mathrm{age}+0.403*\left[\mathrm{AMI}\;\mathrm{location}\;\mathrm{other}\right]\\ & \;\;+0.577*\left[\mathrm{Anterior}\;\mathrm{AMI}\right]+0.468*\left[\mathrm{Previous}\;\mathrm{AMI}\;\mathrm{history}\right]\\ & \;\;+0.767*\left[\mathrm{Killip}\;\mathrm{score}\right]-0.077*\min\left(\left[\mathrm{blood}\;\mathrm{pressure}\right],100\right)\\ & \;\;+\;0.018*\mathrm{pulse}. \end{array}$$

**Figure 1.**
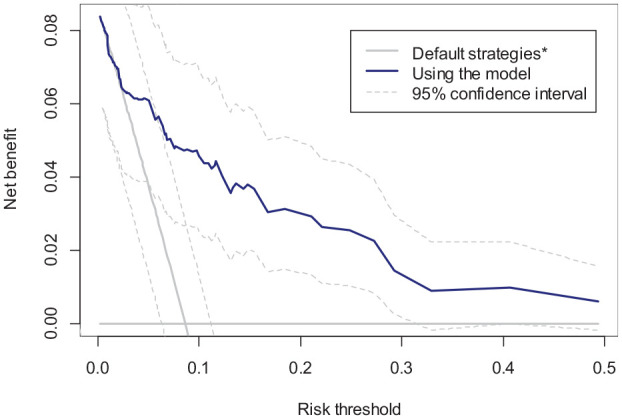
Decision curve (NB) of the candidate model (solid blue), treating all (gray oblique line), and treating none (gray horizontal line) in the validation sample. Bootstrap-based 95% confidence intervals are depicted for the candidate model and treating all strategies (dashed curves). *Treating none (horizontal gray line) and treating all (oblique gray line) NB: net benefit.

The c-statistic of this model is 0.847 in the validation sample. Figure 1 shows the decision curve depicting the empirical NB of the model ( ${\hat{\mathrm{NB}}}_{\mathrm{model}}$ ) alongside those of treating none and treating all. At the 0.02 threshold, the NB of the model was 0.0692, whereas the NB of treating all was 0.0672. As such, based on this validation sample, the use of the model confers a $d\hat{\mathrm{NB}}$ of 0.0020 over the next best decision, which is to treat all.

The gain in NB by using the model over the default strategies can be presented either in terms of change in the number of true positives while holding the number of false positives constant or vice versa.^4^ Here, the difference of 0.0020 means that for every 1,000 treatment decisions, the use of the model will result in an expected gain of 2 true positives. Because a 0.02 threshold implies an exchange rate of 49 between false and true positives, this can also be interpreted as the use of the model resulting in, on average, 2 more true positives × 49 = 98 fewer false positives (i.e., fewer unnecessary treatments) per 1,000 decisions.

However, due to the finite validation sample, a $d\hat{\mathrm{NB}}>0$ does not necessarily mean the model truly confers clinical utility ( dNB>0), and so there is uncertainty in our conclusion that the use of the model is the best strategy at this threshold. Vickers et al.^17^ proposed bootstrapping for inference on NBs. In this scheme, a bootstrap sample from the validation data is obtained, and ${\hat{\mathrm{NB}}}_{\mathrm{model}}$ and ${\hat{\mathrm{NB}}}_{\mathrm{all}}$ are calculated at thresholds of interest. Repeating this step many times provides an empirical distribution for NBs that can be used for constructing confidence intervals around NBs or their differences. Using the percentile method applied to 10,000 bootstraps, the 95% CIs for ${\hat{\mathrm{NB}}}_{\mathrm{model}}$ and ${\hat{\mathrm{NB}}}_{\mathrm{all}}$ are presented as dashed curves in Figure 1. We also obtain a 95%CI for the $d\hat{\mathrm{NB}}$ of −0.0050 to +0.0055. Because the CI crosses zero, the null hypothesis is that dNB=0 cannot be rejected at the 5% significance level. However, the relevance of such hypothesis testing at an arbitrary significance level for decision making is debated.^6,7^ Instead, a risk-neutral decision maker should recommend the model because it has the highest “expected” NB. The impact of any lack of confidence in this recommendation should instead be on whether further research (validation studies) is required.

## A Bayesian Approach toward Interpreting Uncertainties around NB

The conventional bootstrap is akin to assigning a random weight to each observation, with weights drawn from a scaled multinomial distribution. Rubin proposed the Bayesian bootstrap, in which weights are instead generated from a Dirichlet distribution.^18^ They showed that a summary statistic derived from such a bootstrapped sample can be interpreted as a random draw from the posterior distribution of the corresponding population parameter given the sample and a noninformative prior on the underlying data-generating mechanism.^18^ The similarity of the weighting scheme and numerical results between the ordinary and Bayesian bootstrap has resulted in the former being also interpreted in a Bayesian view, as in the approximate Bayesian bootstrap method for the imputation of missing data^19^ or in VoI analysis of cost-effectiveness trials.^20^

**Figure 2:**
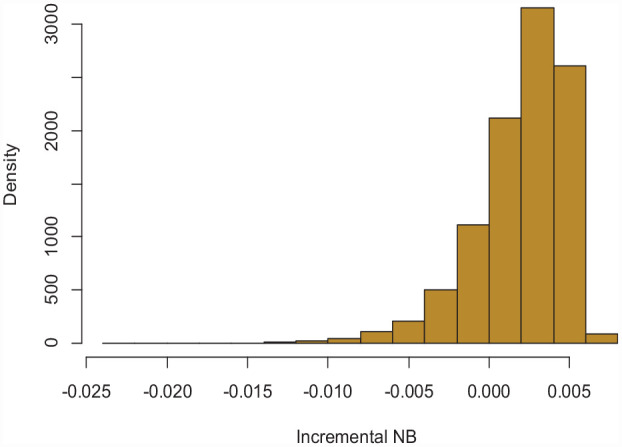
Histogram of the incremental NB of the model (dNB) based on 10,000 bootstraps. NB: net benefit.

Such Bayesian interpretation of the bootstrap enables us to make probabilistic statements about the true NBs. For example, we can count the proportion of bootstraps in which the NB of the model is higher than the NB of the default strategies. This quantity, proposed by Wynants et al.^21^ and termed *P(useful)*, will be the posterior probability that the model-based treatment is truly the strategy with highest NB in the target population. Figure 2 depicts the bootstrap distribution of dNB based on 10,000 bootstraps; *P(useful)*, corresponding to the area that lies on the right side of 0, is 75.9%.

## EVPI

A *P(useful)* < 1 indicates the possibility that our model, despite having the highest expected NB in this validation sample, might not truly be better than the default strategies ( dNB can be negative in the population despite a positive $\hat{\mathrm{dNB}}$ in the sample), in which case the use of the model will be associated with a loss of NB. To quantify the magnitude of this loss, we can follow the steps demonstrated in Table 1. Each row in this table corresponds to a bootstrapped estimate of $NB_{\mathrm{model}},$ $NB_{\mathrm{all}}$ , and $NB_{\mathrm{none}\;(\mathrm{treating}\;\mathrm{no}\;\mathrm{one})}$ , which we can interpret as random draws from the posterior distribution of their true counterparts. In each row, we ask the question, “What decision would have we made, had we known these are the true NBs?” In the first row, $NB_{\mathrm{model}}>NB_{\mathrm{all}}>0$ . As such, using the model remains the best strategy. However, in the second row, $NB_{\mathrm{all}}>NB_{\mathrm{model}}>0$ , so we would have chosen the strategy of treating all. This strategy would give us an NB of 0.1010 (last column), which provides a gain of 0.0074 over the NB of using the model (0.0936), if these were the true NBs.

**Table 1:** Stepwise Calculations for the Expected Gain with Perfect Information for the Case Study^a^

Iteration	$NB_{\mathrm{none}}$	$NB_{\mathrm{model}}$ ^b^	$NB_{\mathrm{all}}$	Winning Strategy	NB of the Winning Strategy
1	0	0.0830	0.0801	Use the model	0.0830
2	0	0.0936	0.1010	Treat all	0.1010
3	0	0.0787	0.0784	Use the model	0.0787
…					
10,000	0	0.0704	0.0654	Use the model	0.0704
**Average**	**0**	**0.0694**	**0.0674**	Use the model: **75.9%**^c^ Treat all: **24.1%** Treat none: **0%**	**0.0699**

NB, net benefit.

a Each row pertains to a random draw from the posterior distribution of $P\left(NB_{\mathrm{model}},NB_{\mathrm{all}}\right)$ , the posterior distribution of true net benefits ( $NB_{\mathrm{none}}=0$ by default). For each row, we find the optimal strategy as the one with the highest NB. The last column records the NB of the winning strategy compared with the use of the model.

b The current strategy is to use the model because it has the highest expected NB among all 3 strategies (the last row).

c Corresponding to a *P*(*useful*) of 75.9%.

Across the bootstraps, the average of ${\hat{\mathrm{NB}}}_{\mathrm{model}}$ is 0.0694, higher than both 0 ( $NB_{\mathrm{none}}$ ) and the average of ${\hat{\mathrm{NB}}}_{\mathrm{all}}$ (indeed, the averages of ${\hat{\mathrm{NB}}}_{\mathrm{model}}$ and ${\hat{\mathrm{NB}}}_{\mathrm{all}}$ across the bootstraps converge to their corresponding estimate in the original sample as the number of bootstraps grows). Thus, in our Bayesian approach, using the model is the strategy with the highest NB based on the current evidence. However, in 24.1% of rows, we would have declared treating all as the winning strategy had we known the true NBs, and in doing so, we would have gained clinical utility over the use of the model. We do not know which value of NBs will emerge as the truth, but we can combine all scenarios by averaging the NB of the winning strategy, which evaluates to 0.0699 (the average of last column values in Table 1). This NB is higher than the NB of using the model by 0.0005. This difference is the expected NB loss due to the finite validation sample or the expected NB gain by knowing the true NBs, a term that we call the *expected value of perfect information* (EVPI) for model validation.

Formalizing the above derivations, we quantify the EVPI by contrasting the expected NBs of the decision-making process under 2 scenarios of current information (estimating NBs with uncertainty in the sample) and perfect information (knowing the true NBs). With the validation sample at hand, the best we can do is to choose the strategy with the highest expected NB, an approach that confers an expected NB of $\max\left\{0,E\left(NB_{\mathrm{model}}\right),E\left(NB_{\mathrm{all}}\right)\right\}$ , with the expectation being with respect to $P\left(NB_{\mathrm{model}},NB_{\mathrm{all}}\right)$ . On the other hand, if we know the true values of NBs, we could choose the most beneficial strategy with certainty, an approach that would confer an NB of $\max\{0,NB_{\mathrm{model}},NB_{\mathrm{all}}\}$ . We do not know the true value of NBs, but we can quantify the expected value of such an optimal strategy given our current knowledge, which would be $E\left\{\max\{0,NB_{\mathrm{model}},NB_{\mathrm{all}}\}\right\}$ , with the expectation being, again, with respect to $P\left(NB_{\mathrm{model}},NB_{\mathrm{all}}\right)$ . The validation EVPI is the difference between these 2 terms:

$$EVPI=E\left\{\max\{0,NB_{model},NB_{all}\}\right\}-\max\left\{0,E\left(NB_{model}\right),E\left(NB_{all}\right)\right\}.$$

## How Can We Interpret the EVPI?

EVPI is a nonnegative scalar quantity in the same units as the NB of risk prediction models, with higher EVPI values indicating higher expected NB loss due to uncertainty. Given that the EVPI is in NB units, the consequence of uncertainty can be presented in the same way as the results of the decision curve analysis. An EVPI of 0.0005 indicates that removing uncertainty about which strategy is the most beneficial is associated with an expected gain of 0.5 in true positives or avoiding an expected 24.5 false positives (unnecessary treatments), for every 1,000 treatment decisions.

Theoretically, any EVPI > 0 indicates the potential value of future validation studies. However, a very low positive EVPI value would indicate a low yield from such a study. A given value of EVPI cannot be declared low or high without considering the decision context. The EVPI measures the expected NB loss per treatment decision due to uncertainty, a loss that is potentially avoidable by performing more validation studies. The true magnitude of this avoidable loss is affected by the number of times the decision of interest is being made in the target population. For example, more than 800,000 AMIs occur every year in the United States,^22^ and a guideline panel in charge of making a national-level recommendation for AMI treatment can consider our candidate model potentially applicable to all such events. In this case, the impact of validation uncertainty is equal to missing the proper intervention in 400 true-positive cases (patients with AMI who will die within 30 d) or imposing unnecessary treatments to 19,600 false-positive cases (patients who will survive) per year. Given such consequences, procuring more samples to reduce this avoidable loss seems justifiable.

An alternative way to contextualize an EVPI value, applicable when the model has the highest expected NB in the validation sample, is the previously proposed relative EVPI.^9^ The relative EVPI compares the expected NB gain with perfect information with the expected NB gain due to use of the model with current information. In our case study, the incremental NB of the model over the next best decision (treating all) is 0.0020. With perfect information, we expect to gain an extra NB of 0.0005. Thus, we can gain, on average, 0.0025/0.0020 = 1.25, or 25% more efficiency by removing validation uncertainty. Such relative EVPI can thus be defined as^9^

$$\mathrm{rEVPI}=\frac{E\left\{\max\{0,NB_{\mathrm{model}},NB_{\mathrm{all}}\}\right\}-\max\left\{0,E\left(NB_{\mathrm{all}}\right)\right\}}{\max\left\{0,E\left(NB_{\mathrm{model}}\right),E\left(NB_{\mathrm{all}}\right)\right\}-\max\left\{0,E\left(NB_{\mathrm{all}}\right)\right\}}.$$

## Computation Algorithms

### (Bayesian) bootstrapping

As explained earlier, a Bayesian interpretation of the bootstrap enables us to use this method readily for EVPI calculations. In this scheme, we interpret $\left({\hat{\mathrm{NB}}}_{\mathrm{model}},{\hat{\mathrm{NB}}}_{\mathrm{all}}\right)$ from a Bayesian or ordinary bootstrapped sample as a random draw from $P\left(NB_{\mathrm{model}},NB_{\mathrm{all}}\right)$ . This in turn enables the calculation of both terms for EVPIs via straightforward Monte Carlo simulations. This results in the algorithm for EVPI presented in Table 2.

**Table 2:** Bootstrap-Based EVPI Computation

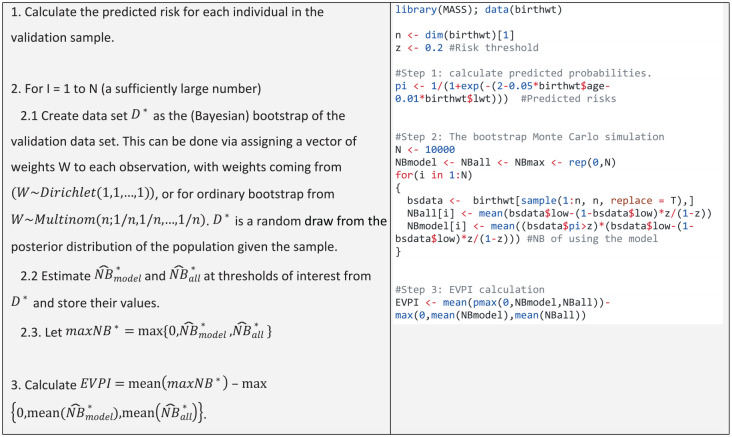

* The R code in the table can be downloaded from https://github.com/resplab/papercode/tree/main/voipred_ex/StylizedExamples.

In calculating the expected NB under current information, we propose to estimate $E(NB_{\mathrm{model}})$ and $E(NB_{\mathrm{all}})$ by, respectively, $\mathrm{mean}\left({\hat{\mathrm{NB}}}_{\mathrm{model}}^{*}\right)$ and $\mathrm{mean}\left({\hat{\mathrm{NB}}}_{\mathrm{all}}^{*}\right)$ , that is, the average of NBs across the bootstrapped samples. Indeed, these quantities converge to the point estimates of their counterparts in the original sample ( ${\hat{\mathrm{NB}}}_{\mathrm{model}}$ and ${\hat{\mathrm{NB}}}_{\mathrm{all}}$ ) as the number of Monte Carlo iterations increases. The use of bootstrap-averaged values over original sample estimates, however, has some advantages. First, this approach prevents getting occasional negative EVPIs due to Monte Carlo noise. Second, if the process of calculating predicted risks has stochastic components (e.g., imputation of missing predictors), this approach enables the incorporation of uncertainty from such components in EVPI calculations. Finally, this approach facilitates the incorporation of prior information via applying rejection sampling to the bootstrap.^23^

### Asymptotic approach based on central limit theorem

Marsh et al.^24^ proposed an asymptotic Wald-type inferential method for NB based on deriving the first 2 moments of the sample distribution of the scaled ${\hat{\mathrm{NB}}}_{\mathrm{model}}$ ( ${\hat{\mathrm{NB}}}_{\mathrm{model}}$ divided by outcome prevalence). They showed that the performance of this method, in terms of the coverage of the resulting confidence interval, is similar to that of the bootstrap-based method, with the advantage of it being a closed-form estimator. We modify their derivation from scaled NB to unscaled NB and from specifying a univariate normal distribution for $NB_{\mathrm{model}}$ to a bivariate normal (BVN) distribution for $NB_{\mathrm{model}}$ and $NB_{\mathrm{all}}$ :

$$\left(NB_{\mathrm{model}},\;NB_{\mathrm{all}}\right)\sim \mathrm{BVN}\left(\left[{\hat{\mathrm{NB}}}_{\mathrm{model}},\;{\hat{\mathrm{NB}}}_{\mathrm{all}}\right],\sum \right),$$

with covariance matrix ∑ having the following components:

$$\begin{array}{c} \mathrm{var}\left(NB_{\mathrm{model}}\right)=\frac{1}{n}\{P_{\mathrm{True}\;\mathrm{Positive}}\left(1-P_{\mathrm{True}\;\mathrm{Positive}}\right)\\ +{\left(\frac{z}{1-z}\right)}^{2}P_{\mathrm{False}\;\mathrm{Positive}}\left(1-P_{\mathrm{False}\;\mathrm{Positive}}\right)\\ +2\left(\frac{z}{1-z}\right)P_{\mathrm{True}\;\mathrm{Positive}}P_{\mathrm{False}\;\mathrm{Positive}}\};\;\\ \mathrm{var}\left(NB_{\mathrm{all}}\right)={\left(\frac{1}{n\left(1-z\right)}\right)}^{2}P_{0}\left(1-P_{0}\right);\\ cov\left(NB_{model},NB_{all}\right)=\frac{1}{n\left(1-z\right)}\left(\left(1-P_{0}\right)P_{True\;\;Positive}+\frac{z}{1-z}P_{0}P_{False\;\;Positive}\right). \end{array}$$

In estimating the above quantities, $P_{0}$ , $P_{\mathrm{True}\;\mathrm{Positive}}$ , and $P_{\mathrm{False}\;\mathrm{Positive}}$ are replaced by their sample estimates. With this parameterization, the first term on the right-hand side of the EVPI equation corresponds to a 2-dimensional unit normal loss integral, for which Lee et al.^25^ recently derived a closed-form solution. The computation steps are provided in Table 3.

**Table 3:** Asymptotic Method for EVPI Eomputation

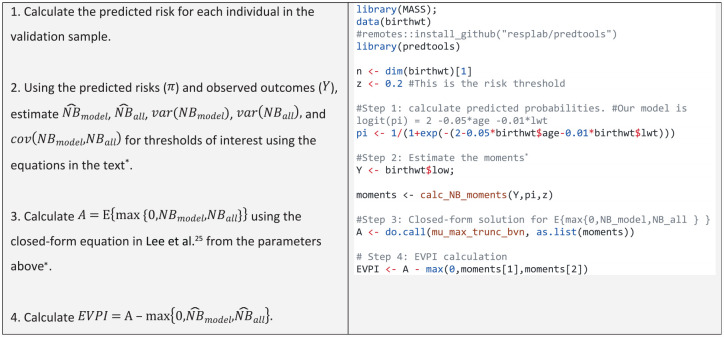

* The *calc_NB_moments* and *mu_max_trunc_bvn* functions are available in the *predtools* R package. The development version can be installed from Github (https://github.com/resplab/predtools). The R code in the table can be downloaded from https://github.com/resplab/papercode/tree/main/voipred_ex/StylizedExamples.

One limitation of this closed-form solution is that it cannot currently be extended to situations in which more than 1 prediction model is considered, as the corresponding truncated (M+1) -variate-normal integral might not have a closed-form solution, requiring numerical integration.^26^

**Figure 3:**
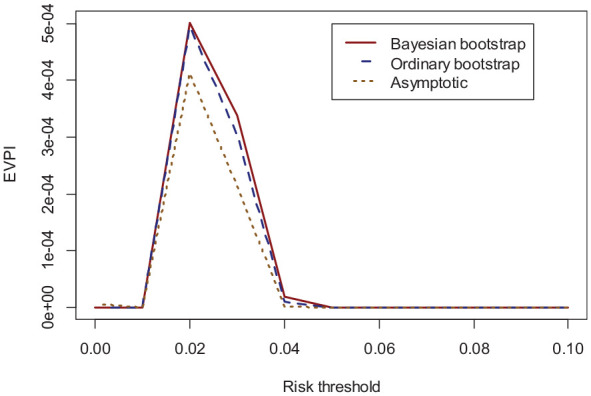
Validation EVPI for the case study as a function of thresholds* for the Bayesian bootstrap (solid red), ordinary bootstrap (dashed blue), and asymptotic method (dotted orange). *Only 3% of predicted risks were at higher thresholds. *EVPI:* Expected Value of Perfect Information.

Figure 3 demonstrates the EVPI values calculated using the Bayesian bootstrap (solid red), ordinary bootstrap (dashed blue), and asymptotic (dotted orange) methods across the (0–0.1) threshold (higher thresholds were considered clinically irrelevant). The asymptotic method for EVPI calculations resulted in an EVPI of 0.0004 at the 0.02 threshold for our case study, while the bootstrap methods both generated an EVPI of 0.0005.

## Brief Simulation Studies

We conducted proof-of-concept simulations, the purpose of which were 2-fold: to evaluate the consistency of computing EVPI using the proposed algorithms and to study how the EVPI changes with validation sample size.

In the first set of simulations, we considered a simple continuous predictor X with a standard normal distribution and assumed the outcome-generating mechanism being of the form logit(P(Y=1|X))=−1.55+0.77X . This choice of coefficients creates a scenario in which the outcome prevalence is 20% and the correct model for Y has a c-statistic of 0.70 (values that we consider typical in validation studies). We first assumed the prediction model happens to be equal to the correct model (returning the correct conditional probability of Y given X ) in the external validation population. We evaluated the EVPI at 3 exemplary thresholds of 0.1 (low). 0.2 (middle, equal to outcome prevalence), and 0.3 (high). This model has an NB of 0.1176, 0.0575, and 0.0270 at these thresholds. The sample size of the validation sample was varied from 250 to 2,000, with doubling in each step.

**Figure 4:**
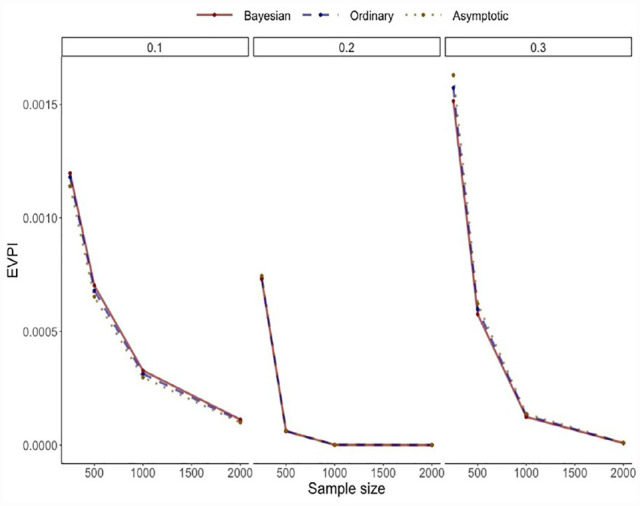
Results of the simulation study for the simple model. *Results are based on 10,000 Monte Carlo simulations. The maximum Monte Carlo Standard Error was 4.45E-06, which is 0.03% of the length of the Y-axis. Within each simulation, EVPIs were calculated with 1,000 simulations for the bootstrap-based methods. *EVPI:* Expected Value of Perfect Information.

Figure 4 provides the results, which are the average of 10,000 simulations. The 3 computation algorithms generated nearly identical results. As expected, the EVPI declined with larger sample sizes, with an expected pattern of diminishing gains with large samples.

Supplementary Material Section 1 provides the results of related simulation scenarios in which the prediction model was changed in different ways (change in discrimination via adding an error term to predictions, and change in calibration by perturbing the model intercept). The results demonstrate the particularly strong effect of model miscalibration on EVPI values, with striking nonlinearities between EVPI and model calibration that varied by the risk threshold. On the other hand, there was generally an inverse association between c-statistic and EVPI (detailed explanations are provided in Supplementary Material).

**Figure 5:**
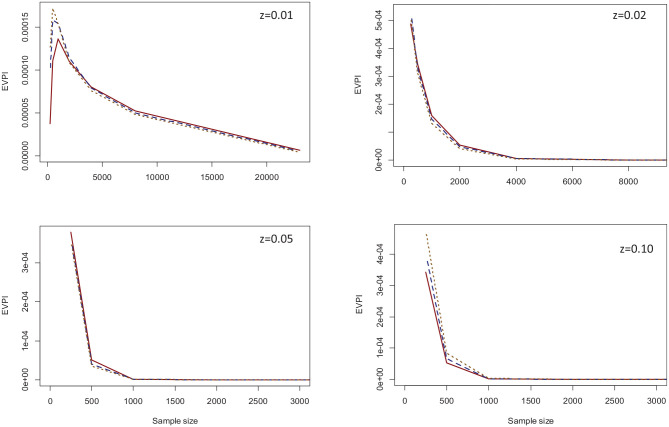
EVPI values across the range of sample sizes for the three computation methods and threshold values of 0.01 (top left), 0.02 (top right), 0.05 (bottom left), and 0.10 (bottom-right). Solid red: Bayesian bootstrap; dashed blue: ordinary bootstrap; dotted orange: asymptotic method.* *The X-axis for three of the panels is truncated as the higher values were all zero. Results are based on 1,000 Monte Carlo simulations. *EVPI:* Expected Value of Perfect information.

The second set of simulations was related to the case study. We simulated larger validation samples by using an increasingly larger subset of the US sample of GUSTO-I as the external validation data set. We repeatedly (1,000 times) drew samples without replacement from the entire validation sample, starting from *n* = 250 and doubling it in each step to the maximum size (23,034). We investigated the EVPI at 4 thresholds of 0.01, 0.02, 0.05, and 0.10. Figure 5 provides the results on how EVPI changes as a function of sample size for the GUSTO-I study.

Again, all 3 methods generated similar results. For all thresholds except z = 0.01, the EVPI declined with increasing sample size, with the decline being very steep for higher threshold values that were close to outcome prevalence (0.05 and 0.10). With the full validation sample, the EVPI was 0.000005 for *z* = 0.01 and 0 for other thresholds. The only nonintuitive finding was for *z* = 0.01 at small sample sizes, where the EVPI increased when the sample size increased from 250 to 500 for all 3 computation methods and from 500 to 1,000 for the Bayesian bootstrap method. Of note, the 0.01 threshold is significantly lower than outcome prevalence. Only 13% of observations in the original validation sample had a predicted probability below this threshold, and none had an event. Because of this, the sample variance of dNB is 0 in many Monte Carlo iterations, resulting in EVPI = 0 and *p*(*useful*)=1 (see discussion for interpretation).

## Discussion

In this work, we defined the validation EVPI as the expected loss in NB due to the finiteness of an external validation sample and the associated risk of incorrectly identifying the optimal strategy. We developed algorithms based on bootstrapping and asymptotic methods for EVPI computation. In proof-of-concept simulation studies, we showed EVPI calculation algorithms generated consistent results, and EVPI generally behaved as expected, in that with more information (larger sample size) it declined, but very large sample sizes were associated with diminishing gain. Simulations showed that validation EVPI was particularly sensitive to model calibration. We interpreted the EVPI in a clinical example for predicting risk of mortality after heart attack. An R implementation of the proposed method is provided in the *predtools* package (https://github.com/resplab/predtools/).

Decision curve analysis has been considered a breakthrough in predictive analytics as it enables the estimation of decision-theoretic metric (NB) for risk prediction models. However, whether and how to quantify uncertainty around DCA remains controversial.^6,7^ Principles of decision theory stipulate that for a risk-neutral decision maker and in the absence of (substantial) irrecoverable costs associated with a change in practice, the decision whether to adopt a model for clinical practice should be based on its expected NB in comparison with the expected NB of the default decisions, irrespective of uncertainty.^27^ From this perspective, uncertainty around NB informs whether, independently of the decision concerning the adoption of the model, future validation is required.^27^ Given the interpretability of EVPI, we propose presenting EVPI values in decision curve analysis.

We proposed interpreting the EVPI based on scaling it to the population, as well as comparing it to the NB gain associated with the use of the model. In cost-effectiveness analysis where the payoffs can be converted to net monetary benefit, VoI metrics are typically in monetary units.^28^ Thus, when scaled to the population, VoI metrics can be compared with the budget of research to objectively inform whether future empirical studies are justifiable.^28^ However, such calculations involve considering all relevant costs and health consequences of competing strategies over a sufficiently long time horizon, an approach that most often warrants decision-analytic modeling.^29^ A main appeal of the NB approach in risk prediction is that it uses the information in the validation sample, without having to obtain often jurisdiction-specific evidence or making assumptions on long-term consequences of interventions. While we advocate for such full decision modeling in its due course (e.g., after an impact study has quantified the resource use associated with implementing the model at point of care), the EVPI proposed here involves much fewer assumptions and generates results that can be interpreted alongside the results of decision curve analysis. As such, it has the potential to become a standard component of validation studies and provide general guidance on the impact of uncertainty due to the finite validation sample.

Our previously proposed development EVPI captures the expected NB loss due to the distance between the correct (strongly calibrated) model and a candidate model developed using a finite sample.^9^ Its computation requires modeling P(Y|X) to characterize the distribution of the calibrated risks. Such explicit modeling was not required in the validation EVPI algorithms. This is because the correct model needs not be specified when the goal is to determine if a prespecified model performs better than default strategies. However, modeling might be warranted when the effective sample size is small. This was demonstrated in one of the simulation scenarios with GUSTO-I data, in which the low threshold and small sample size resulted in counterintuitive findings (EVPI declining with a larger sample). The EVPI is directly affected by the var(dNB) , whose sample estimate can be 0 in small samples and extreme thresholds. VoI analysis in such situations might require a full Bayesian approach to incorporate prior beliefs. In general, however, the suitability of the available sample in such instances should be carefully considered before embarking on the external validation of a model.

There are several directions for future research in applying VoI to clinical prediction models. Dedicated simulation studies are required to compare more deeply the performance of the EVPI computation methods. A distinct area of future research is to develop a framework for expected value of sample information (EVSI) analysis for development and validation of prediction models. While EVPI puts an upper bound on the expected NB gain with more information, EVSI represents the expected gain in NB associated with an empirical study of a given specification (e.g., sample size) and can thus more specifically guide future research. Further, treatment benefit models that predict an individual’s response to a specific treatment are gaining momentum in predictive analytics,^30,31^ and VoI methods that estimate the expected gain with further development or validation of such models should be developed.

The proposed validation EVPI is applicable to the decision curve analysis for a typical external validation study in which a prespecified model is evaluated in a single new population. A broader context is multicenter studies, in which hierarchical Bayesian methods can be used to model differences among settings.^13,21^ An important decision in such settings is whether to use 1 global model across all settings or whether setting-specific models should be developed^32^. The finite sample results in NB loss in from different sources: uncertainty in the structure and coefficient of setting-specific models (related to development VoI) and uncertainty in the decision to use global versus setting-specific models (related to validation VoI). In such hierarchical settings, the expected NB loss quantity therefore has elements of both development and validation EVPI, and its development should be pursued separately.

Uncertainty is a fact of life during all stages of predictive analytics including development, validation, implementation, and revision of clinical prediction models. Such uncertainty is conventionally quantified and communicated using classical inferential metrics. VoI combines the probability of incorrect decisions due to uncertainty with the expected loss in clinical utility into a single measure and therefore provides a complete picture of the consequences of uncertainty.^8^ VoI metrics, now available for the development and validation phases of risk prediction modeling, have the potential to provide actionable insight on the need for further evidence across the life cycle of predictive algorithms.

## Supplemental Material

sj-pdf-1-mdm-10.1177_0272989X231178317 – Supplemental material for Value-of-Information Analysis for External Validation of Risk Prediction Models Click here for additional data file. Supplemental material, sj-pdf-1-mdm-10.1177_0272989X231178317 for Value-of-Information Analysis for External Validation of Risk Prediction Models by Mohsen Sadatsafavi, Tae Yoon Lee, Laure Wynants, Andrew J Vickers and Paul Gustafson in Medical Decision Making
