# Supplementary material for: Value-of-Information Analysis for External Validation of Risk Prediction Models
Source: Med Decis Making. 2023 Jun 22;43(5):564–75. doi: 10.1177/0272989X231178317 (PMC10336716; doi:10.1177/0272989X231178317)
Supplement: sj-pdf-1-mdm-10.1177_0272989X231178317 – Supplemental material for Value-of-Information Analysis for External Validation of Risk Prediction Models [file sj-pdf-1-mdm-10.1177_0272989X231178317.pdf]

Supplementary Material for

"Value of Information Analysis for External Validation of Risk Prediction Models"

Section 1: Proof-of-concept simulations on the relation between EVPI and sample size and model characteristics

The validation EVPI is the expected loss in net benefit (NB) associated with the risk of incorrectly identifying which strategy (using the model, treating all, or treating none) is the most beneficial based on a finite validation sample. In the main text, we showed that validation EVPI generally declines with increasing sample size. In this section, we further investigate how EVPI changes as a function of the performance characteristics of the model, defined in terms of its calibration and discrimination.

The model

We model a stylized scenario, as described in the main text, in which there is only one predictor, X, which has a standard normal distribution in the target (validation) population. The true data generating function for the binary outcome Y is of the typical logistic form

$$logit\big(P(Y=1|X)\big) = \beta_0 + \beta_1 X.$$

We chose $\beta_0 = -1.55$ and $\beta_1 = 0.77$ to reflect a 'typical' scenario: the event probability in the population is 0.2, and the best prediction model, which is the true outcome generating function itself, has a c-statistic of 0.70.

Changing discrimination (and calibration slope)

This was achieved by introducing noise to the predicted risks. This in turn was modeled by adding a normally distributed random variable (with zero mean and varying standard deviation [SD]) to the logit of the predicted probabilities. Adding such a noise will result in random changes in the ranking of predicted risks, thus reducing model discrimination. Because the variance of predicted risks generated in this way exceeds that of the true risk, this setup simulates optimistic predictions, manifested as the calibration slope being less than one. We tested scenarios with error SDs in {0.15, 0.30, 0.45, 0.60}, corresponding to c-statistics of, respectively, 0.695, 0.685, 0.671, and 0.657, and calibration slopes of, respectively, 0.96, 0.86, 0.73, and 0.60.

Changing calibration intercept while preserving discrimination

This was performed by creating prediction models that are only different in the intercept (β_0) from the actual outcome probabilities. This approach preserves the c-statistic of the model (0.70) but changes its calibration intercept. We applied differences of {-1.6, -0.8, -0.4, 0.4, 0.8, 1.6} to the intercept. We note that at extreme negative values, the model will severely underestimate the correct risks, and its use is effectively equal to treating none. Reciprocally, at extreme positive values, the model will severely overestimate the correct risks, and its use is effectively equal to treating everyone.

Analysis

EVPIs were evaluated at three risk thresholds of 0.1 (lower than prevalence, thus the model competes with treating all), 0.2 (at prevalence, where the NBs of treating all and none are both zero), and 0.3 (higher than prevalence, thus the model competes with treating none).

EVPIs were produced for sample sizes in {250,500,1000,2000} using the Bayesian bootstrap, ordinary bootstrap, and asymptotic methods. The bootstrap-based methods were based on 1,000 bootstraps. Each analysis was performed by averaging 10,000 independent simulations.

Results

Figure S1 demonstrates the EVPI of the perturbed models as a function of model discrimination. ***Figure S2*** rearranges the same results as a function of the calibration intercept. When evaluating EVPI as a function of sample size, in all scenarios EVPI changed in the expected direction (lower EVPI values with higher sample size).

The EVPI monotonically increased when model discrimination was degraded (***Figure S1***). This is expected because in all these simulations the model remains moderately calibrated(1), and it is known that as long as a model is calibrated, $NB_{model} \geq max\{NB_{all}, 0\}$(2). This can be interpreted as more uncertainty associated with the superiority of a model with degraded performance (lower c-statistic). Here, with degraded discrimination, dNB gets closer to 0 but remains non-negative. With smaller values of dNB, we become less confident around the superiority of the model, so EVPI increases accordingly.

Figure S1: EVPIs at three exemplary thresholds as a function of the SD of prediction noise (X axis) and sample sizes (colored lines) for three calculation methods (column) at three exemplary threshold (rows)

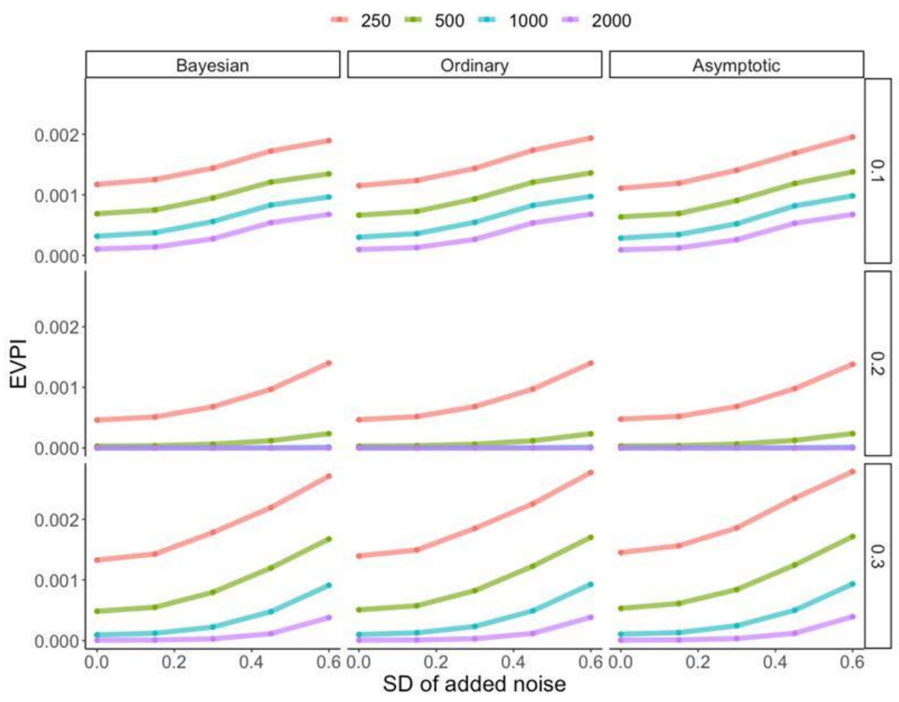

EVPI: Expected Value of Perfect Information

On the other hand, the EVPI as a function of the calibration intercept of the model was non-monotonical and highly dependent on the risk threshold. Two different patterns are observed here depending on if the threshold was equal to the outcome prevalence (middle row) or away from it (first and last rows).

EVPI as a function of model intercept when risk threshold is away from outcome prevalence (first and third rows)

When the model is calibrated, EVPI is low because there is little uncertainty about the superiority of the model over the default strategies. On the other hand, at extremes of model miscalibration, EVPI approaches zero. This is because the NB of the model is negative and the EVPI effectively becomes about the uncertainty between the two default decisions:

$$EVPI \ = \mathrm{E}\{max(NB_{all}, 0)\} - max(\mathrm{E}(NB_{all}), 0\}.$$

At thresholds away from outcome prevalence, on the left treating all dominates treating none, and on the right treating none dominates treating all. In both instances, the distribution of NB_{all} does not cover 0, and the two term on the right-hand side of the above equation are equal, resulting in EVPI=0. The behavior of EVPI in between is non-monotonical and depends on the direction of miscalibration and whether risk threshold is above or below the outcome prevalence.

EVPI as a function of model intercept when risk threshold is equal to outcome prevalence

The EVPI here has its smallest value when calibration intercept is 0. This is because at this threshold, the model has the highest dNB and there is little uncertainty in the superiority of the model. However, as the calibration is degraded, its dNB gets close to 0 and EVPI increases. At higher level of miscalibration, dNB becomes negative. In such instances, there will be little uncertainty in the inferiority of the model, and the EVPI effectively becomes about the uncertainty between the two default decisions:

$$EVPI \ = \mathrm{E}\{max(NB_{all}, 0)\} - max(\mathrm{E}(NB_{all}), 0\}.$$

But a crucial point is that the NB curves of the two default strategies cross at this risk threshold. At this threshold, where $\mathrm{E}NB_{all} = 0$, there is substantial uncertainty about which of the two default strategies is truly zero; thus the two term on the right hand side of the above equation are not equal.

Figure S2: EVPIs at three exemplary thresholds as a function of calibration intercept (X axis) and sample sizes (colored lines) for three calculation methods (column) at three exemplary threshold (rows)

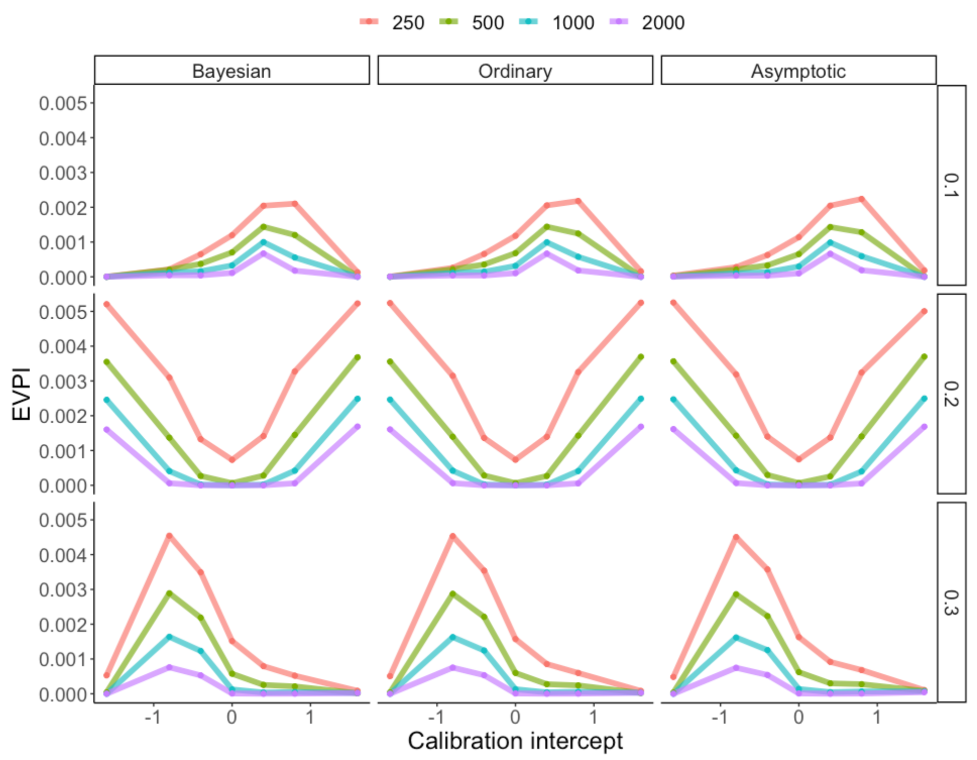

EVPI: Expected Value of Perfect Information

References

1. Van Calster B, Nieboer D, Vergouwe Y, De Cock B, Pencina MJ, Steyerberg EW. A calibration hierarchy for risk models was defined: from utopia to empirical data. J Clin Epidemiol. 2016;74:167–76.

2. Van Calster B, Vickers AJ. Calibration of risk prediction models: impact on decision-analytic performance. Med Decis Mak Int J Soc Med Decis Mak. 2015 Feb;35(2):162–9.
